# High-Resolution X-ray Phase-Contrast Imaging and Sensory and Rheometer Tests in Cooked Edamame

**DOI:** 10.3390/foods11050730

**Published:** 2022-03-01

**Authors:** Masafumi Hidaka, Shuhei Miyashita, Naoto Yagi, Masato Hoshino, Yukiya Kogasaka, Tomoyuki Fujii, Yoshinori Kanayama

**Affiliations:** 1 Graduate School of Agricultural Science, Tohoku University, Aoba-ku, Sendai 980-8572, Japan; masafumi.hidaka.a4@tohoku.ac.jp (M.H.); shuhei.miyashita.d7@tohoku.ac.jp (S.M.); atom@tohoku.ac.jp (T.F.); 2 Scattering and Imaging Division, Japan Synchrotron Radiation Research Institute, Hyogo, Sayo 679-5198, Japan; yagi@spring8.or.jp (N.Y.); hoshino@spring8.or.jp (M.H.); 3 JA Sendai, Miyagino-ku, Sendai 983-0039, Japan; yukiya_kogasaka@jasendai.or.jp

**Keywords:** X-ray phase-contrast computed tomography, 3-D imaging, edamame, vegetable soybean

## Abstract

Although several reports exist on the use of X-ray analysis in vegetables and fruits to examine internal disorders, cavities, and porosity, information on X-ray analysis of qualities, such as texture, is lacking as well as information on X-ray analysis of legumes. Therefore, this study aimed to perform X-ray analysis with sensory and rheometer tests in cooked vegetable soybean (edamame). Edamame is popular worldwide due to its deliciousness and nutritional value. Vascular structures and cracks around them were clearly visualized using X-ray phase-contrast computed tomography (CT) imaging. In addition, we observed the fine structure of the seed coat, which could be important for seed development, germination, and processing. The density in the edamame beans declined as the boiling time increased, promoting a reduction in hardness described in sensory and rheometer tests. The reduction in density proceeded from the gap between cotyledons, the opposite side of the hypocotyl, and the crack. Collectively, the findings show that the high-resolution X-ray phase-contrast CT imaging conducted in a nondestructive manner may help in effectively evaluating the quality of vegetables and in observing the internal structures related to plant development.

## 1. Introduction

With respect to the quality of vegetables and fruits, sugars [1,2], functional ingredients [3], and minerals [4] related to flavor and nutrition are important. Furthermore, texture is one of the most important criteria to evaluate quality. One method for evaluating the quality of vegetables and fruits is a sensory evaluation test, which effectively evaluates the complex food quality. However, because of limitations in terms of cost, sample volume, and throughput, rheological analysis is used along with a sensory evaluation test to evaluate the texture [5,6]. Texture is a multi-parameter attribute derived from the structure of foods, indicating the importance of the internal structure [7]. A multidisciplinary collaboration between different fields is necessary to evaluate complex food quality, including texture [5]. X-ray analysis can be considered an additional approach to the conventional methods for evaluating the internal food structure. The upper limits of the amount of X-ray irradiation to food and leaked X-ray doses are legally set, and at the practical stage, developing analytical methods and equipment at a level that does not affect foods and operators is required. 

Non-destructive internal structural analysis using X-rays is one technique for evaluating the quality of vegetables, fruits, and nuts. According to a review by Schoeman et al. [8], nondestructive analysis using X-rays helps avoid food loss by detecting internal disorders and facilitates the study of the relationship between food quality and internal structures, including at the cellular level. For example, internal disorders in apples are evaluated by visualizing and quantifying them at high resolution using X-ray microcomputed tomography (CT) [9]. Moreover, X-ray CT has helped in elucidating the movement of gas and water during storage in eggplants, turnips, apples, and pears by internal structure analysis, particularly porosity mapping [10]. These previous reports contain a relatively high number of findings on internal disorders, cavities, and porosity from X-ray analyses; however, studies of texture in vegetables and fruits seem to be rare. Information on X-ray analysis of legumes is also lacking. In addition, despite one study on X-ray phase-contrast CT for microstructure changes due to coffee bean processing [11], few studies have used this method. 

Edamame, or vegetable soybean, is popular both in Japan and internationally because of its deliciousness and nutritional value [12,13]. The effects of cultivar and storage on the quality of edamame have been assessed using sensory evaluation tests [13,14,15]. A previous report also evaluated the texture characteristics of different cultivars by combining sensory evaluation analysis with instrumental analysis [6,12,16]. On the other hand, reports assessing the effects of processing and cooking methods are rare, and to the best of our knowledge, there have been no reports on the use of X-ray analysis to evaluate the quality of edamame. Therefore, it is meaningful to evaluate the quality of edamame by sensory and rheometer tests and to examine the usefulness of a new technique in X-ray analysis in relation to the cooking method. Compared to conventional radiographs, phase-contrast X-ray imaging can greatly enhance the visibility of soft, living tissues [17,18].

Therefore, we propose the method using X-ray phase-contrast CT as a methodological approach to evaluating the quality of edamame showing different textures in sensory and rheometer tests. As reported above, the use of X-ray phase-contrast CT has been shown to be a valuable as a methodological approach in analyzing the effect of the processing method on the microstructure of coffee beans [11]. The phase-contrast X-ray imaging technique used by these authors was based on the propagation and refraction of X-rays. However, the Talbot method (a grating-based technique) may also be used because it detects phase changes more sensitively and provides a higher-density resolution. We also examined the effect of the cooking method on the quality of edamame based on the structural changes. Our study has potential significance both in establishing evaluation methods using X-ray analysis that can be used for processing, cooking, breeding, and cultivation and in developing a method to elucidate the mechanisms of quality formation through visualizing the internal structure using X-ray as a nondestructive analytical method.

## 2. Materials and Methods

### 2.1. Sample Preparation

Edamame-type soybean (*Glycine max* cv. Miyagi-shirome) cultivated in Sendai (Miyagi prefecture, Japan) was used for the study. Within one day post-harvest, the pods were blanched (primary heating) for two minutes in boiling tap water. The blanched beans were removed from the pods and stored for several months at −18 °C. This procedure follows one of Japan’s major commercial processing conditions of edamame beans. For further tests and experiments, the frozen beans were thawed in running tap water, boiled (secondary heating) in 4% salt solution for different periods (2, 6, or 15 min), and chilled immediately in wet ice. As the volumes of boiling water and chilling wet ice used were more than 30 times those of the beans, the beans reached the target temperature rapidly.

### 2.2. Sensory Evaluation Tests and Rheological Analysis

A panel comprising 26 members of Tohoku University (ages 21–59 years, 12 females, all non-smokers, all had previously consumed edamame) evaluated the processed beans. Most panelists had not been trained for affective tests but were pre-instructed in both how and what to evaluate. The panel evaluated the following 11 components: four mechanical characteristics (hardness, elasticity, stickiness, and crumbliness), two taste characteristics (sweetness and umami intensity), four preference components (biting texture, feel on the tongue, taste, and smell), and total preference. These components were listed based on some earlier studies [6,13,16]. Mechanical and taste characteristics were scored in an objective manner—whether the intensity of each characteristic was either low or high—and preference components and total preference were scored in a subjective manner—whether individual panelists liked or disliked it. For all the evaluation components, beans boiled for six minutes were always given a standard score (i.e., 3), and the panelists scored the others (i.e., those boiled for two minutes and 15 min), in comparison with those boiled for six minutes, between 1 (low or dislike) and 5 (high or like). 

We tested processed beans for mechanical characteristics using a creep meter RHEONER II (Yamaden, Japan) equipped with a cylindrical plunger (1 mm diameter) as described previously [19]. We removed the seed coats of the beans and fixed either one of the cotyledons to the stage, as the plunger penetrated the center of the cotyledon from the outer abaxial side, and obtained strain–stress profiles. We set the plunger speed to 1 mm per second.

Statistical analyses were performed with R software version 4.1.2 [20] with the “exactRankTests” package version 0.8-34 [21]. The statistical analysis method used for each dataset is indicated in the figure captions and results section.

### 2.3. X-ray Phase-Contrast CT

We measured unprocessed and processed beans using X-ray phase tomography based on X-ray Talbot interferometry located at the bending magnet beamline BL20B2 at SPring-8, Japan [22,23,24,25]. A monochromatic X-ray beam was fine-tuned to 20 keV and passed in sequence through a nickel phase grating (G1) with a pattern thickness of 3.53 µm and a gold absorption grating (G2) with a pattern thickness of 20 µm. Both transmission gratings had a pitch size of 2.4 µm and a pattern size area greater than 30 × 30 mm^2^. Moiré fringe patterns produced by X-ray beams passing through the sample and the two gratings were detected using a visible-light conversion type X-ray detector comprising a beam monitor AA60 (Hamamatsu Photonics) and a high-definition CMOS camera (C13949-50U, Hamamatsu Photonics). The effective pixel size was 3.47 µm. G2 was shifted relative to G1 with a Piezo stage using a five-step fringe-scan method for phase retrieval. Samples were embedded in agarose gel to prevent motion. During measurements, each sample was fixed on a rotator, creating 1000 projection images. Integration of the differential phase images produced by the scans created the final phase images used for tomographic reconstruction. The images were reconstructed using an in-house software developed at SPring-8. Reconstructed cross-sections were obtained in 16-bit TIFF images. A pixel value in the image represents the amount of phase shift in each pixel. Image processing was performed with ImageJ [26] and Fiji [27].

### 2.4. Absorption Based X-ray CT

X-ray CT experiments were conducted at the beam line BL14B2 of SPring-8. The X-ray wavelength was tuned at 1 Å using a double Si-crystal monochromator (net plane: (111)). The specimen was set on a turntable, following which 360 X-ray transmission images were obtained by rotating the specimen stepwise at a step of 0.5 degree throughout 0–180 degrees. The exposure time for each image acquisition was 250 ms. An X-ray imaging system consisting of AA40 X-ray imaging unit and C4880-41S CCD camera (Hamamatsu Photonics K.K) was used for the image acquisition. Pixel size and frame size of the image data was 2.92 × 2.92 µm^2^ and 3200 × 1200 pixels, respectively. The distance from the specimen to the X-ray camera was set at 30 mm. The intensities of pixels in the transmission images were converted into X-ray transmission rate of the specimen by dividing the intensities by those of an associated image of incident beam without the specimen. From these converted image data, we reconstructed a stack of tomograms, which indicate the distribution of linear X-ray absorption coefficient in the specimen, by the filtered back projection method.

## 3. Results

### 3.1. Sensory Characteristics and Rheological Analyses of Edamame Beans

**Figure 1:**
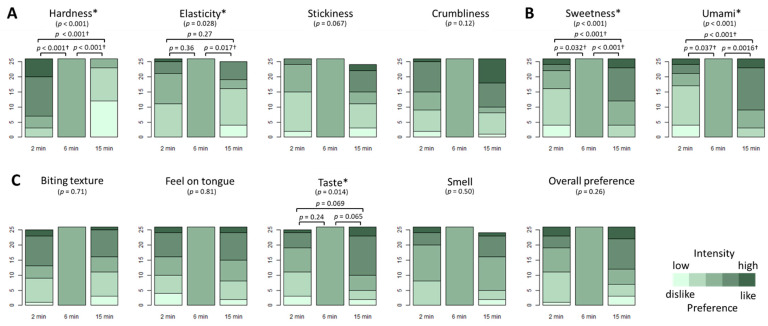
Sensory characteristics of edamame beans boiled for 2, 6, and 15 min. Objective evaluations for physical characteristics (**A**) and taste components (**B**) and subjective evaluation for individual preferences (**C**) by 26 panelists are shown by cumulative bar plots. Asterisks (*) indicate the evaluation components where significant differences among processing conditions were observed (Friedman test, *p* < 0.05); for these components, pairwise comparisons between any two of the three combinations were performed, and the combinations with significant differences are marked by daggers (†) (Wilcoxon signed-rank test with Holm correction at α = 0.05).

We prepared edamame beans boiled for 2, 6, and 15 min in 4% salt solution, subsequent to two minutes’ blanching and storage in a freezer. They were first tested for sensory characteristics. Of the four textural characteristics tested (Figure 1A), we found significant differences among the three different processing conditions for the scores for hardness and elasticity (Friedman test, *p* < 0.05). Pairwise comparison revealed that hardness decreased continuously from 2 min to 6 min and from 6 min to 15 min, whereas elasticity decreased from 6 min to 15 min (Wilcoxon signed-rank test, applying Holm correction with α = 0.05). The two taste characteristics, sweetness and umami, both showed significant differences related to boiling conditions (Friedman test, *p* < 0.05), which could be explained by their continuous increase from 2 min to 6 min and from 6 min to 15 min (Figure 1B). In respect of preference components and overall preference, we detected a significant difference only for taste preference (Friedman test, *p* < 0.05); however, we did not detect this significant difference in pairwise comparisons between any two processing conditions. We found no correlation to sex for any of the 11 components (*p* = 0.21~1.00, data not shown).

**Figure 2:**
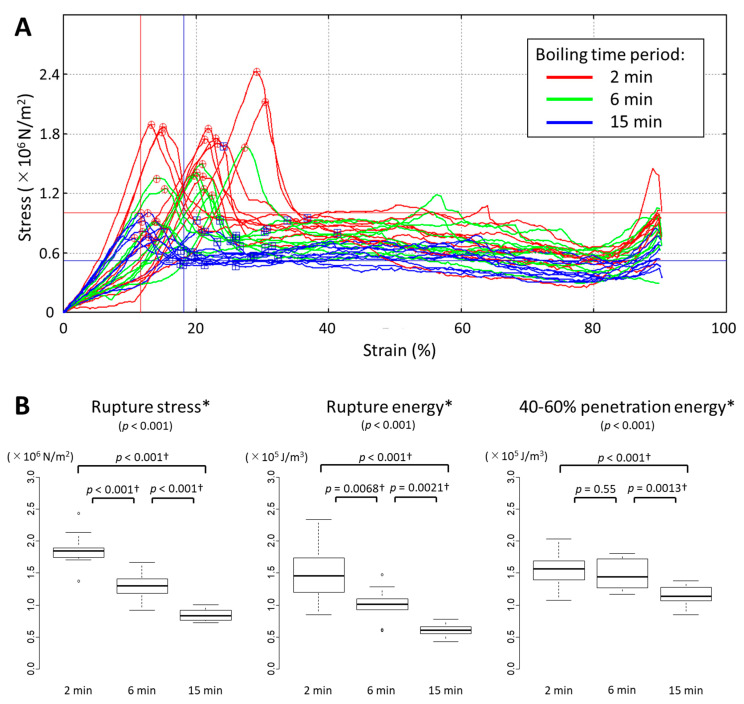
Plunger penetration tests of edamame beans boiled for 2, 6, and 15 min. Strain–stress profiles of the penetration of edamame beans by a cylindrical plunger (1-mm diameter) are shown in (**A**). Rupture stress, rupture energy, and 40–60% penetration energy are shown by box plots (**B**). Non-parametric statistical analyses were applied because of the heteroscedasticity of the data. Asterisks (*) indicate the measurements that showed significant differences among processing conditions (Kruskal–Wallis test, *p* < 0.05). Daggers (†) indicate the combinations that showed significant difference between two processing conditions (Wilcoxon rank-sum test with Holm correction at α = 0.05). White circles (°) indicate outliers.

To further examine the textural aspects of edamame beans processed in different ways, we used a creep meter to analyze the plunger penetration characteristics of ten independent cotyledons per each processing condition. The strain–stress profiles are shown in Figure 2A, and the measurement data for rupture stress, rupture energy, and penetration energy between 40% and 60% strains are summarized in Figure 2B. All three measurements displayed significant differences among the three processing conditions (Kruskal–Wallis test, *p* < 0.05). The results of pairwise comparisons indicated that rupture stress and rupture energy declined continuously between 2 min and 6 min boiling and between 6 min and 15 min boiling, whereas there was a significant decline in penetration energy only between 6 min and 15 min (Wilcoxon rank-sum test, applying Holm correction with *α* = 0.05).

### 3.2. Visualization of Microstructure of Edamame Beans by X-ray Phase-Contrast CT

**Figure 3:**
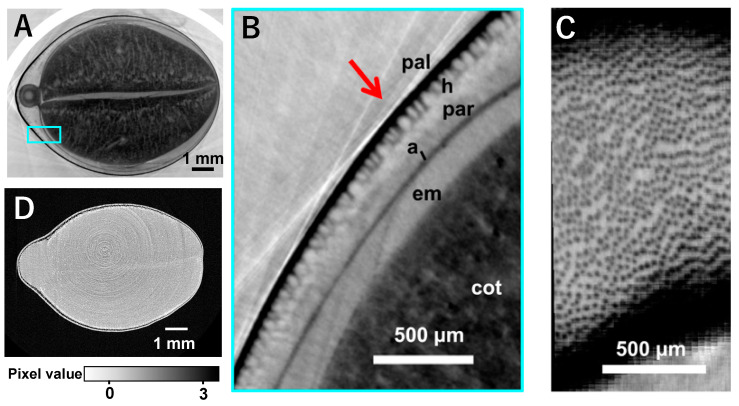
Images of X-ray phase-contrast CT (**A**–**C**). An X-ray absorption image (**D**) was taken for comparison with X-ray phase-contrast, and other images, whose pixel value represents phase shift, were prepared by X-ray phase-contrast. A tomogram of an edamame bean (**A**,**D**), high-resolution image (3.47 μm/pixel) of the epidermis of the edamame bean for the area indicated by the cyan square in **A** (**B**), and the cross-sectional image of the hourglass cell layer perpendicular to the red arrow in **B** (**C**). The black dots are assumed to be hourglass cells (**C**). Abbreviations in (**B**): pal, palisade layer; h, hourglass cell; par, parenchyma; a, aleurone cells; em, endosperm; cot, cotyledon.

We observed unprocessed edamame beans by X-ray phase-contrast CT (Figure 3A–C). Figure 3A is a tomographic image of the cross section slightly above the middle of the cotyledon, and the upper and lower cotyledons and the gap between them were observed with the hypocotyl on the left. Figure 3B shows a high-resolution image of the blue frame area in Figure 3A. Palisade layer, hourglass cells, and aleurone cells were observed as reported by Miller et al. [28]. The layer of hourglass cells observed in Figure 3B is displayed as a cross-section perpendicular to the red arrow (Figure 3C), and it is likely that the hourglass cells shown as black dots at a certain distance were visualized. Figure 3D shows an X-ray absorption image taken for comparison with X-ray phase-contrast CT; internal structure was barely observable by this method.

**Figure 4:**
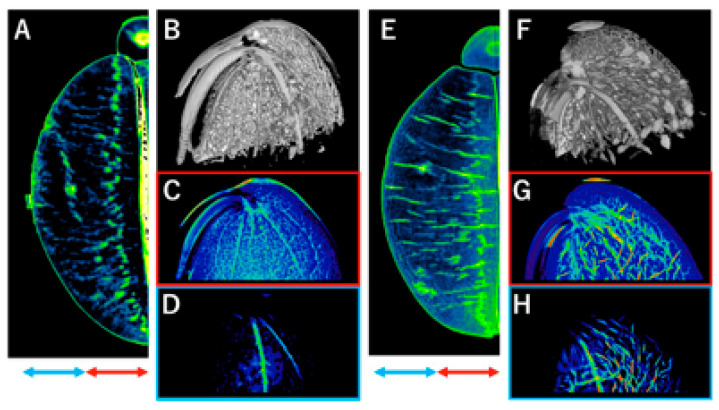
Three-dimensional and projection images of edamame beans. Visualization of vascular tissue with images (**B**,**F**) reconstructed in the low-density portion of the cotyledon (**A**,**E**). The projection images were reconstructed in the parts near to (indicated by the red, double-headed arrow, (**C**)) and far from (indicated by the blue, double-headed arrow, (**D**)) the gap between the cotyledons in A, and similarly, the projection images (**G**,**H**) were reconstructed in the parts near to and far from the gap between the cotyledons in (**E**). The images were created by projection in the direction of the double-headed arrows. The average values of the pixel intensities are projected, and lower integrated values in red indicate lower density. Blue, yellow, and red indicate high-, intermediate-, and low-density values, respectively. Materials that were not boiled and materials that were boiled for 2 min were used in (**A**–**D**) and (**E**–**H**), respectively.

The image was reconstructed in the low-density part of the cotyledon as seen in Figure 4A, and the vascular tissues were visualized as shown in Figure 4B. Figure 4C is the projection image of the section near the gap between the cotyledons specified by the red, double-headed arrow in Figure 4A (inside), and Figure 4D is the projection image of the section far from the gap specified by the blue, double-headed arrow in Figure 4A (outside). We observed many vascular tissues in the “inside” region (Figure 4C), whereas we detected only two thick, vascular tissues that extended outward (“outside”, Figure 4D). Similar images were reconstructed for the beans boiled for two minutes (Figure 4E–H, which correspond to Figure 4A–D (non-boiled), respectively). As seen in Figure 4E, in the 2D image, many cracks appear in the direction perpendicular to the gap between the two cotyledons. As seen in Figure 4F, these cracks were observed as discs and appeared to be derived from the vascular tissues. Moreover, in the projection image of “inside” of Figure 4E (Figure 4G), we detected cracks as low-density red and also observed cracks in the “outside” (Figure 4H).

### 3.3. Density Distribution in Edamame Beans by X-ray Phase-Contrast CT

**Figure 5:**
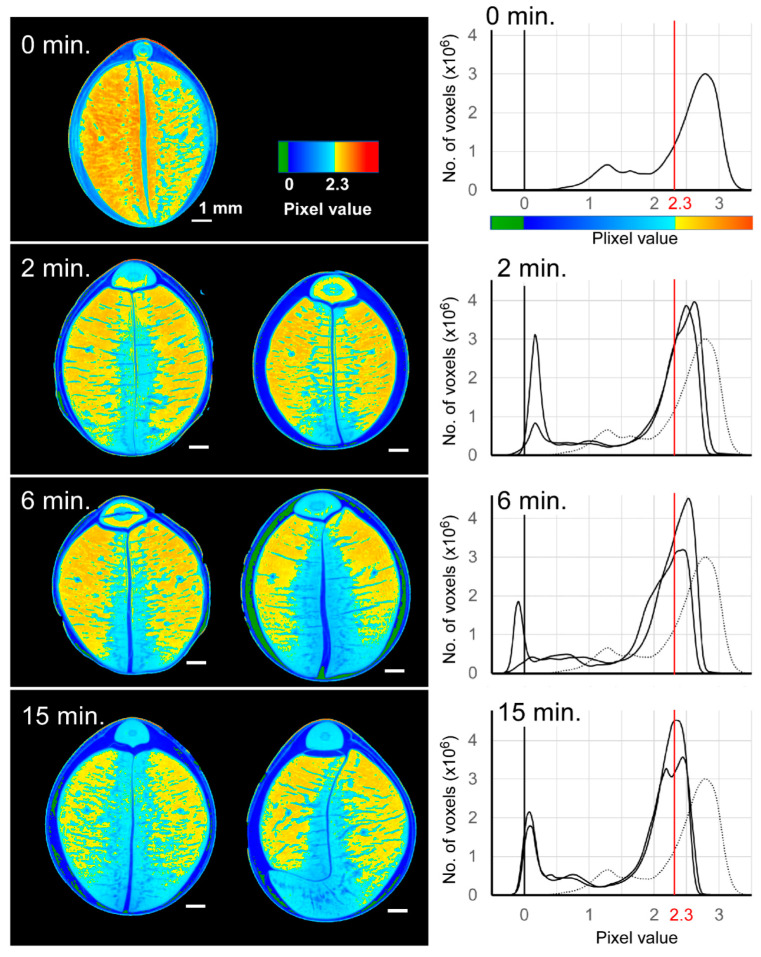
Tomogram images of edamame beans with different boiling times. (**Left**); Two samples are shown for boiling times of 2, 6, and 15 min. (**Right**); Frequency distribution of the pixel value of each voxel. In each histogram, the distribution of non-boiled samples is shown by the broken line.

We imaged in 2D changes in density distribution over time (Figure 5). The blue region with a density with a pixel value of 2.3 or lower increased with the boiling time, and it spread from the gap between the cotyledons, particularly as seen from the bottom of each picture. Moreover, the parts we assumed to be cracks also appeared to be involved in the spread of the low-density regions. The density distribution graph in Figure 5 shows that the peak position was higher than pixel value of 2.3 at 0 min of boiling time, shifted with the increase of boiling time, and showed a pixel value of 2.3 at 15 min. In addition, the high-density portion of pixel value > 2.3 and its ratio per volume decreased as boiling time increased (Supplementary Table S1). The small peak around the pixel value of 1.2 at 0 min in Figure 5 was in the seed coat part, and the low-density peak shifted to approximately the water value with a pixel value of 0 by boiling.

## 4. Discussion

We performed an evaluation using X-rays of edamame, focusing on the significance of the development of the method for evaluating the vegetable quality. In the sensory evaluation tests, the physical characteristics and taste components displayed variations in hardness, elasticity, sweetness, and umami depending on the boiling time. In previous reports, different cultivars exhibited observed differences either in hardness, sweetness, and umami [6,16] or in sweetness and chewability, which may be related to hardness and elasticity [13], implying that these evaluation criteria are important for both comparisons between cultivars and between cooking methods. Regarding the subjective evaluation, we found no difference in taste in the pairwise comparison and no difference in the overall preference, indicating difficulties for subjective analysis compared with analyzing physical characteristics and taste components. This finding suggests that individual preferences varied considerably among the panelists even though the same panelists could identify clear differences in some objectively evaluated components. Furthermore, the results of the subjective test were analyzed by dividing the overall preferences into high and low scores (Supplementary Figure S1). These findings indicate that variations in individual preferences could be attributed to both textural and taste preferences despite the panelists reacting differently to many of the same components. In the future, devising a method to acquire data from each evaluation method and present integrated results by multivariate analysis is expected. 

We conducted an instrumental-based analysis to indirectly assess the texture. Flores et al. [12] found weak but significant correlations between sensory evaluation tests and texture analyzer analysis in the comparison between cultivars. Similarly, in this study, the hardness value obtained from the sensory evaluation test and the rupture stress and rupture energy values obtained from the rheological analysis displayed the same tendencies, indicating a relationship between the sensory evaluation tests and instrumental analysis. The findings further showed that the significant difference in the elasticity found in the sensory evaluation tests corresponded to a penetration energy of 40–60%. Thus, despite being a destructive, the rheological analysis uses many parameters and is thus valuable in quality evaluation. Our findings are valuable because there is little knowledge about the correlation between rheological and sensory evaluation in edamame and evaluation of the effect of the heating time. Here, X-ray analysis was attempted using materials with observed differences in quality through sensory testing and rheometer analysis. Although rheometer data can indicate the quality of edamame, the internal structure should be observed by nondestructive X-ray analysis to understand the mechanisms involved in quality differences. X-ray analysis can provide 2D density images and 3D internal structure images and shows potential as a new method for evaluating quality. 

In this study, we observed the fine structure of edamame and visualized the vascular tissues. The advantages of phase-contrast X-ray imaging over absorption X-ray imaging include excellent observation of the internal structure of plant stems [29], and this is supported by our findings (Figure 3 and Figure 4). Regarding vascular tissues, embolism of the grape axis has been reported observed by 3D images of X-ray CT [30]. More recently, 2D images have reportedly observed laurel embolism by X-ray micro-CT [31]. Besides embolism, 2D images of the vascular tissues of soybean leaf have been reported [32]. Nevertheless, 3D imaging of the vascular tissues of fruits, seeds, and beans is scarce, particularly clear imaging with high resolution. Mahajan et al. [33] reported observation of the vascular tissue in soybean seeds for testing viability and vigor related to seed germination rate by fluorescence imaging, not by X-ray analysis, and the image was unclear. Moriwaki et al. [34] reported magnetic resonance imaging (MRI) of the vascular tissues of a fleshy fruit. There are some interesting MRI reports on plant tissues [35,36], in which not only the structure of plant tissues but also their water status can be observed. Consequently, MRI should be compared with X-ray analysis in relation to heating as described later. It has been reported that the hilum region, including the vascular tissue, is involved in hydration in common beans [37]; thus, the relationship between the vascular tissues and the cracks formed around the vascular tissues in this study could be interesting. Our findings show that the X-ray phase-contrast CT, without fixation and contrast media, enables one to image the intact internal structure and observe vascular tissues with high resolution. This method would be suitable not only for evaluating food quality but also for analyzing diseases and physiological disorders related to vascular tissues.

In this study, we were also able to observe the fine structure of seed coats. Seed coats are involved in the development, dormancy, germination, and defense of seeds as well as in cooking and processing [38]. Thus, seed coats are important both in plant science and in food science. The prevalence of studies focusing on seed coats is such that more than 7000 documents arise when searching with Scopus using “seed coats” as a keyword. Optical and electron microscopic observations of seed coats in previous studies [28,38] necessitate fixation and sectioning. Conversely, X-ray phase-contrast CT, as attempted in this study, could be useful because it allowed clear observations to be made in a non-destructive manner. Developing a method for observing the seed coat is important because hourglass cells specifically accumulate soybean peroxidase and could be used as “phytofactories” for protein production [38]. The results of this study show well-imaged characteristic structures of hourglass cells, such as columnar styles, and the arrangement at regular intervals.

Detection of internal cracks in legumes has been reported in both common beans [39] and soybeans [33]; both were performed for testing viability and vigor related to the seed germination rate, which is not for food science research, and screened at low resolution. Compared with the previous reports, we detected clear cracks at higher resolutions. Moreover, various methods have been devised for observing cracks, including tomography, projection, and 3D imaging. The results of observation indicated water-infiltration routes and density-reduction patterns that may affect the changes in texture due to the brittleness of the tissues. Therefore, we anticipate that the methods we have reported here will contribute to understanding the mechanisms of quality change and identifying its indicators. Furthermore, although the resolution is relatively lower, MRI is an effective method for imaging the water status [35,36]; therefore, our findings can be confirmed by combining MRI with X-ray phase-contrast CT.

With regards to the processing method and X-ray CT, a report exists in which X-ray phase-contrast CT of coffee bean microstructure was used to evaluate the porosity of the beans before and after roasting [11]. Although not involving soybeans, as an example of the evaluation of beans, the effect of microwave treatment on the cooking and macronutrient qualities has been reported via Fourier transform mid-infrared spectroscopy [40]. However, non-destructive analyses of the relationship between vegetables containing edamame and heating time remains limited. In this study, the image of density reduction due to the heating of edamame likely corresponded to the results of sensory evaluation tests and rheological analysis, suggesting one aspect of the mechanism of texture change. The reduction in density is likely related to the infiltration of water; based on 2D and 3D image data and the parameters obtained, the hilum region, including the vascular tissue in beans, is reported to be involved in water infiltration in germination and processing [37]. Therefore, it is possible that water infiltrates through the vascular tissues from the hilum region and causes the formation of cracks. Moreover, water infiltration from either the micropyle or the seed coat region is also conceivable. In that case, water can easily enter the seed coat region and the space between cotyledons from an early stage and may then infiltrate through vascular tissues and cracks. In particular, we found more vascular tissues on the “inside” near the space between cotyledons than on the “outside”, which is considered to be the cause of density reduction from the inside. As the cell structure of edamame is maintained under some heating [41], water infiltration routes were likely affected by the tissue structure, and an uneven density was observed in this study. The same group has reported that when the cell structure is maintained, the difference in pear fruit texture is due to the cell wall [42]; therefore, a similar analysis is necessary for edamame.

## 5. Concluding Remarks

According to a review of food X-ray CT [8], X-ray CT research focuses mainly on internal disorders, cavities, and porosity. Analyses of microstructures, such as the present study, appear to be rare. Information on legumes is also lacking in this field. We conducted evaluations by X-ray phase-contrast CT using cooked edamame, showing different textures in sensory and rheometer tests and determined the significance of the method. In the future, this method can be advanced to practice by increasing the number of samples, quantifying density and cracks in the X-ray analysis data, and analyzing the correlations between the evaluation methods. As an example of using the X-ray analysis data for quality evaluation, the correlation between the rupture stress (in which the same difference was observed as hardness in the sensory test) and the high-density ratio (Supplementary Table S1) in the X-ray analysis was examined, and a correlation of 0.83 was obtained (*p* < 0.05, data not shown). In this study, the number of samples was limited for conducting high-resolution analysis. However, if clarification of the structure of the observation target and the required resolution can be achieved, many samples can be measured simultaneously. Furthermore, as few reports examining microstructures by non-destructive analysis exist, the findings of this study can be expected to contribute not only to the evaluation of food quality but also to plant science. Since it was difficult to visualize the complicated internal structure of edamame with simple X-ray CT, the advantage of using X-ray phase-contrast CT became clear. Moreover, in the analysis by section preparation, only analysis data on the prepared surface can be obtained. However, X-ray phase-contrast CT enables a large amount of data to be obtained in one analysis, and repeated image analysis and numerical evaluation for all angles and positions are possible by reconstructing 2D and 3D images. Because few studies using similar analytical methods exist in which the internal structure in vegetables and fruits can be visualized at high resolution without fixation or contrast media, our results may apply to agricultural products in addition to edamame.

## Supplementary Materials

The following supporting information can be downloaded at: https://www.mdpi.com/article/10.3390/foods11050730/s1, Figure S1: Analyses of preference data subsets filtered by overall preference of each panelist; Table S1: Effect of boiling time on phase shift indicated by pixel value in the cotyledons and hypocotyls of edamame.
