# Supplementary material for: High-Resolution X-ray Phase-Contrast Imaging and Sensory and Rheometer Tests in Cooked Edamame
Source: Foods. 2022 Mar 1;11(5):730. doi: 10.3390/foods11050730 (PMC8909405; doi:10.3390/foods11050730)
Supplement: Supplementary file 1 [file foods-11-00730-s001.zip › foods-1590400-supplementary.pdf]

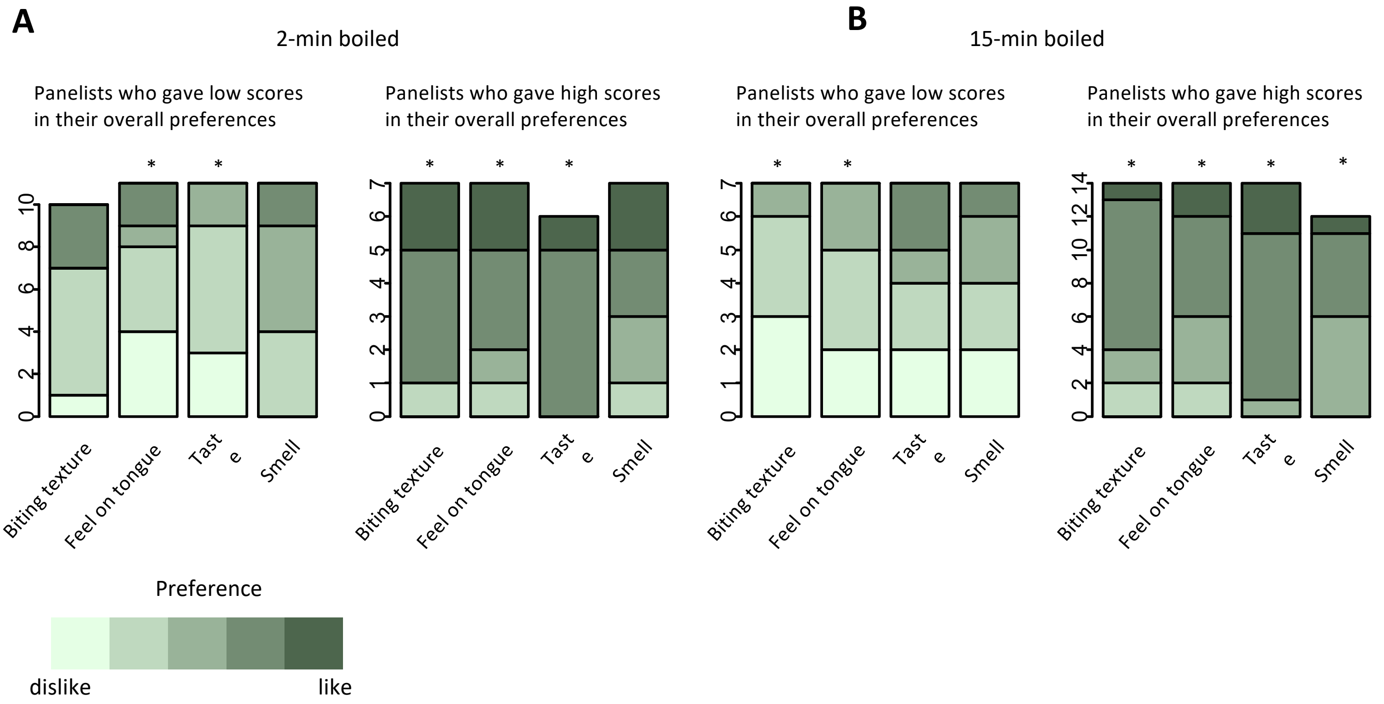

Supplementary Figure S1. Analyses of preference data subsets filtered by overall preference of each panelist. The dataset was filtered by the overall preference of each panelist; i.e., whether the panelist gave a high or a low score to edamame beans boiled for 2 minutes (A) and 15 minutes (B) compared with ones boiled for 6 minutes. Preference components were further analyzed to determine which of the components explain the overall preference. Asterisks indicate the preference components that showed significant difference from edamame beans boiled for 6 minutes (Wilcoxon signed-rank test with Holm correction at $\alpha = 0.05$). Pairwise comparisons revealed that the panelists who gave low scores to edamame beans boiled for two minutes gave significantly low scores for "feel on tongue" and "taste" (Wilcoxon signed-rank test, $p < 0.05$), suggesting that the negative overall preference could be attributed to these factors. The panelists who gave high scores to edamame beans boiled for two minutes gave significantly high scores for "biting texture," "feel on tongue," and "taste". The panelists who showed negative preference for edamame beans boiled for 15 minutes gave significantly low scores for "biting texture" and "feel on tongue," whereas those who displayed a positive preference gave significantly high scores for all four components.

Supplementary Table S1. Effect of boiling time on phase shift indicated by pixel value in the cotyledons and hypocotyls of edamame.

Boil time	Total voxels	(Volume; mm^3)	No. of high density voxels (pixel value > 2.3)	High density ratio
0 min.	48,934,194	(130.9)	41,573,544	0.85
2 min.	56,133,193	(150.1)	36,771,575	0.66
2 min.	50,016,360	(133.7)	30,154,519	0.6
6 min.	79,845,698	(213.5)	46,653,101	0.58
6 min.	63,469,177	(169.7)	33,678,705	0.34
15 min.	58,536,775	(156.6)	21,583,421	0.37
15 min.	55,596,266	(148.7)	20,692,475	0.37
